# Identification of key genes and modules in response to Cadmium stress in different rice varieties and stem nodes by weighted gene co-expression network analysis

**DOI:** 10.1038/s41598-020-66132-4

**Published:** 2020-06-12

**Authors:** Qi Wang, Xiannan Zeng, Qiulai Song, Yu Sun, Yanjiang Feng, Yongcai Lai

**Affiliations:** 1 Institute of Crop Cultivation and Tillage, Heilongjiang Academy of Agricultural Sciences, 368 Xuefu Road, Nangang District, Harbin, 150000 Heilongjiang China; 2 Heilongjiang Academy of Agricultural Sciences, 368 Xuefu Road, Nangang District, Harbin, 150000 Heilongjiang China

**Keywords:** Plant signalling, Plant stress responses

## Abstract

Soil cadmium (Cd) pollution threatens food safety. This study aimed to identify genes related to Cd accumulation in rice. Low- (Shennong 315, short for S315) and high- (Shendao 47, short for S47) Cd-accumulative rice cultivars were incubated with CdCl_2_·2.5H_2_O. RNA-seq and weighted gene co-expression network analysis (WGCNA) were performed to identify the modules and genes associated with Cd-accumulative traits of rice. After Cd stress treatment, the Cd content in various tissues of S315 was significantly higher than that of S47. In the stem nodes, the Cd distribution results of the two varieties indicated that the unelongated nodes near the root (short for node A) had a stronger ability to block Cd transfer upwards than the panicle node (short for node B). Cd stress induced huge changes in gene expression profiles. After analyzing the differentially expressed genes (DEGs) in significantly correlated WGCNA modules, we found that genes related to heavy metal transportation had higher expression levels in node A than that in node B, such as Copper transporter 6 (OS04G0415600), Zinc transporter 10 (OS06G0566300), and some heavy-metal associated proteins (OS11G0147500, OS03G0861400, and OS10G0506100). In the comparison results between S315 and S47, the expression of chitinase (OS03G0679700 and OS06G0726200) was increased by Cd treatment in S315. In addition, *OsHSPs* (OS05G0460000, OS08G0500700), *OsHSFC2A* (OS02G0232000), and *OsDJA5* (OS03G0787300) were found differentially expressed after Cd treatment in S315, but changed less in S47. In summary, different rice varieties have different processes and intensities in response to Cd stress. The node A might function as the key tissue for blocking Cd upward transport into the panicle via vigorous processes, including of heavy metal transportation, response to stress, and cell wall.

## Introduction

Heavy metal cadmium (Cd) contaminates a large area of rice (*Oryza sativa*), which is one of the largest food crops in China and worldwide^1^. Cd pollution causes irreversible soil problems in China. Various technologies and great efforts have been made in the treatment of Cd pollution and restoration of Cd contaminated soils, but the little effect has been achieved. Screening and breeding for low Cd-accumulative cultivars could relieve Cd-induced pressure in cropping system^2,3^.

Many plant species with low cadmium accumulation have been proposed and widely promoted in scientific research and cropping^2,4^. Consequently, identifying genetic targets that can be used to reduce cadmium accumulation in crops is important for plant breeding^5–8^. Many genes related to Cd stress have been identified. For instance, the expression of *Arabidopsis* PLANT DEFENSIN 2 (*AtPDF2.5*) can promote Cd accumulation in *Arabidopsis* roots^5^. Tang *et al*. reported that the knockout of *OsNramp5* (a magnesium ion (Mg2+) and Cd transporter and a Mn and Cd uptake gene) in rice reduced the accumulation of Cd in rice^8^.

The comparative transcriptome analysis of low and high Cd accumulation genotypes has been performed on *Brassica Chinensis* L.^9^, wheat^10,11^, rice^12^ and many other crops. Application of molecular and transgenic technologies and next-generation sequencing (NGS) methods facilitate the identification process of effective genes related to Cd fixation and distribution^9–11^, and some genes have been recommended as targets for controlling Cd accumulation in crops^9^. However, the emergence of a large number of proposed targets increased the difficulty of breeding strategy. Weighted gene co-expression network analysis (WGCNA) approach is a popular method used for identifying the co-expressed and hub genes^13,14^. WGCNA has been widely used to identify genes and modules associated with the specific phenotypes and agronomic traits^15,16^.

In production practice, we found two varieties, one with high Cd accumulation (Shennong 315, S315) and one with low Cd accumulation (Shendao 47, S315). Here, we aimed to reveal the differences in response to Cd stress in stem nodes between the two rice varieties using the RNA-seq combined with the WGCNA approach. The transcriptome profiles of the unelongated nodes near the root (node A) and the panicle node (node B) were compared. The results will give us more understanding of the gene expression profiles in responding to Cd stress between different stem nodes and between different rice varieties.

## Results

### Cd content profiles in different tissues of different rice varieties

The results showed that the Cd concentrations in the unelongated nodes near the root (node A), the panicle node (node B) and grains (Gr) of the two rice cultivars significantly increased (*p* < 0.0001, Table 1) after Cd treatment. As expected, *Shennong 315* (S315) was a low grain-Cd-accumulating rice cultivar and *Shendao 47* (S47) was a high one (3.06 ± 0.21 mg/kg in S315; 4.84 ± 0.54 mg/kg in S47) after Cd treatment. In both the two rice cultivars, the Cd concentration of node A (64.26 ± 7.23 mg/kg in S315; 86.43 ± 9.37 mg/kg in S47) were significantly higher than that of node B (5.63 ± 0.90 mg/kg in S315; 19.96 ± 4.23 mg/kg in S47, *p* < 0.001, Table 1). Interestingly, in the control groups, a small amount of Cd was still accumulated in both two rice cultivars, and most of them were enriched in node A (Table 1).

**Table 1:** The Cd concentration in different sites of the two cultivars before and after Cd treatments.

Groups	Control (mg/kg DW)	Cd treatments (mg/kg DW)	P
S315-A	16.24 ± 4.36b	64.26 ± 7.23b	<0.0001
S315-B	1.41 ± 0.02d	5.63 ± 0.90d	<0.0001
S47-A	31.41 ± 5.23a	86.43 ± 9.37a	<0.0001
S47-B	2.17 ± 0.51c	19.96 ± 4.23c	<0.0001
S315-Gr	0.19 ± 0.02 f	3.06 ± 0.21 f	<0.0001
S47-Gr	0.45 ± 0.05e	4.84 ± 0.54e	<0.0001

S47 note high Cd-accumulative cultivar “*Shendao 47*”, S315 notes low Cd-accumulative cultivar “*Shennong 315*”, “A” indicates the first node, “B” indicates panicle node. Gr, grains. P value was tested using the unpaired t test. The different lowercases in the column notes the significant differences (p < 0.05) by the one-way ANOVA test.

### Summary of the mRNA-seq

Illumina transcriptome sequencing produced 1132.54 M clean reads, with an average Q20 and Q30 value of 97.54% and 93.42%, respectively (Supplementary Table 1). The mapping rate to the reference genome (ftp://ftp.ensemblgenomes.org/pub/release-44/plants/fasta/oryza_sativa/) ranges from 82.33% to 96.49%.

### Identification of DEGs related to Cd treatments

By identifying the DEGs between different nodes in the same rice cultivar and DEGs between different rice cultivars in the same node, different effects of Cd treatment on the transcriptome in the two rice cultivars and stem nodes were confirmed (Supplementary Fig. 1). There were 390 and 520 DEGs induced by Cd treatment in node A and B of S315, respectively. And 883 and 306 DEGs found in node A and B of S47 after Cd treatment. GO-BP category enrichment analysis showed that the Cd-induced DEGs in node A and B of the S315 cultivar were mainly associated with “response to heat”, “response to temperature stimulus”, “protein folding” and “transcription regulator activity” (Supplementary Table 2). While in S47 cultivar, no significant enriched GO terms found in node A, and six significant GO terms (“tetrapyrrole binding”, “heme binding”, “oxidation-reduction process” and “defense response”) found in node B (Supplementary Table 2). The KEGG enrichment analysis results showed that “MAPK signaling pathway-plant”, “Signal transduction” and “Environmental Information Processing” were significantly enriched in node A and B of the S315 cultivar and node B of the S47 cultivar. No significantly enriched KEGG pathways found in node A of the S47 cultivar (Supplementary Table 2). To understand the gene expression profile in response to Cd stress, we used Zhou’s^9^ figure as a reference to show the DEGs expression patterns in the rice stem nodes (Fig. 2). The results showed that ABA signal pathway, Glutathione metabolism, and some transporters play important roles in the process of resistance to Cd stress. We found that *OsSnRN2* showed higher expression in S47 than that of S315. The expression of *OsHSF* in S315 was decreased by Cd stress, of which the expression showed no obvious changes in S47. In addition, *OsHSPs* showed a similar expression profile with that of *OsHSF*. As the well-known Cd related transporters, most *OsNRAMP* family members showed no significant differences between control and Cd treatment. Another Cd related transporter *OsHMA3* (OS07G0232900) was significantly increased by Cd treatment in S315, but showed slight changes in S47. In addition, node B had higher expression of GLN and glutathione peroxidase (*OsGSH-Px*) than node A in S315, but the profile changes were not obvious in A, especially the *OsGSH-Px* gene.

**Figure 1:**
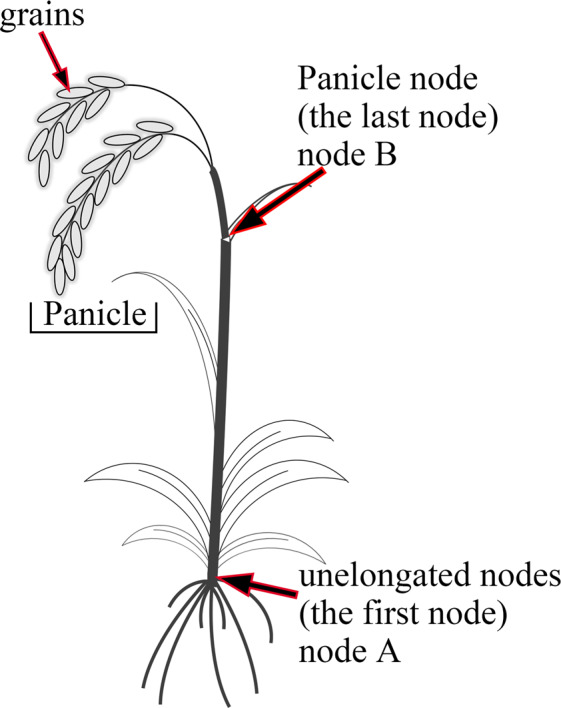
The diagrammatic drawing of rice with indicated sampling sites.

**Figure 2:**
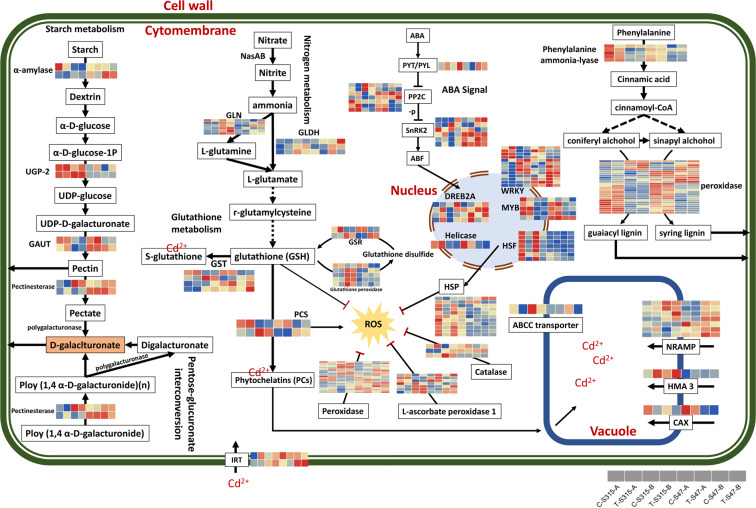
The expression patterns of DEGs response to Cd stress in different groups. Heatmaps shows the expression level of DEGs, and the redder the color, the higher expression. Original figure is from Zhou’s report, and modified according to our results. Original figure from: Zhou, Q. *et al*. Comparative transcriptome analysis between low-and high-cadmium-accumulating genotypes of pakchoi (Brassica chinensis L.) in response to cadmium stress. Environmental science & technology 50, 6485–6494 (2016).

### Identification of co-expression networks associated with rice phenotypes and traits by WGCNA

**Figure 3:**
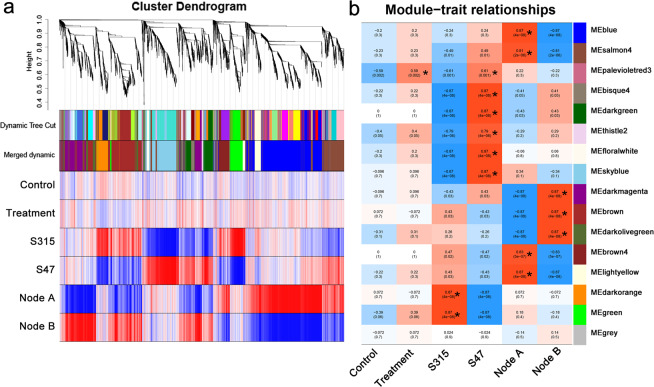
WGCNA module identification and correlation analysis. (**a**) the clustering dendrogram and expression heatmap of genes identifying the WGCNA modules. (**b**) the correlation of the identified modules with the Cd content in different treatments. Modules significantly associated with the traits with identified with |cor | >0.5 and p value < 0.05, and are indicated by asterisk*. Red and blue color notes positive and negative correlation with gene expression, respectively.

In order to identify the DEGs associated with the different phenotypes and traits, all DEGs were pooled into one set and used for WGCNA. A total of 7358 DEGs (from the comparisons of C-S315-A *vs*. C-S315-B, T-S315-A *vs*. T-S315-B, C-S47-A *vs*. C-S47-B, C-S47-A *vs*. C-S315-A, T-S47-A *vs*. T-S315-A, T-S47-B *vs*. T-S315-B, T-S47-A *vs*. T-S47-B, C-S47-B *vs*. C-S315-B, C-S315-A *vs*. T-S315-A, C-S47-B *vs*. T-S47-B, C-S315-B *vs*. T-S315-B, and C-S47-A *vs*. T-S47-A) were pooled and analyzed (Supplementary Table 3). The adjacency matrix parameters, including soft-thresholding power and connectivity between DEGs, were screened prior to the WGCNA module analysis. The soft threshold power of 21 (β = 21) was selected according to the preconditions of approximate scale-free topology (Supplementary Fig. 2). Accordingly, a total of 16 WGCNA modules were identified (Fig. 3a), including 9 to 1743 genes in each WGCNA module. We identified that 1, 8 and 7 modules showed significant (cor > 0.5, p < 0.05) correlation with the Cd content in different Cd treatment, rice cultivar and nodes, respectively (Fig. 3b).

### Modules and DEGs associated with nodes

**Figure 4:**
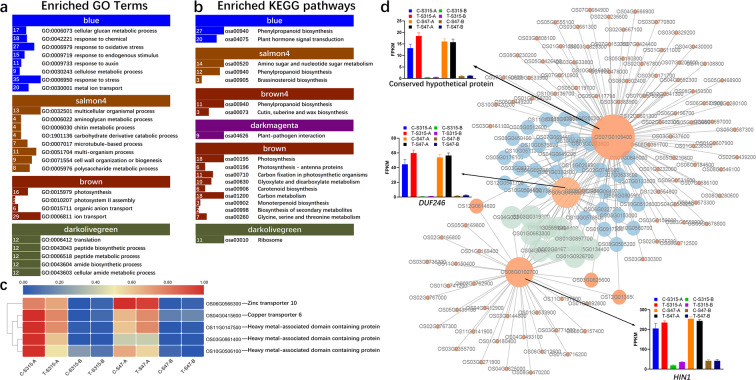
Enrichment of the modules associated with panicle node. The Gene Ontology biological processes (GO-BP, **a**) and KEGG pathways (**b**) associated with the modules relevant to panicle node. The number ahead of the items indicates the gene number. (**c**) the expression of several genes in the node A negative module (blue). (**d**) gene interaction network and key regulator expression. The bigger the node, the greater the number of connections it has. Nodes with different colors are with different number of connections.

Our present study identified the co-expression of genes in 4 modules (blue, salmon4, brown4, and lightyellow) were significantly and positively correlated with the trait of node A (cor > 0.5, p < 0.05), but negatively with that of node B (cor < −0.5, p < 0.05). While 3 modules (darkmagenta, brown, and darkolivegreen) were significantly and positively correlated with the trait of node B (cor > 0.5, p < 0.05), but negatively with that of node A (cor < −0.5, p < 0.05). DEGs in blue module were mainly associated with “response to oxidative stress”, “response to endogenous stimulus”, “response to auxin”, “response to stress”, “metal ion transport” etc. DEGs in salmon4 module were involved in BPs associated with “multicellular organismal process”, “chitin metabolic process”, and “cell wall organization or biogenesis”. While DEGs in brown module were associated with photosynthesis and Cd transport. And DEGs in darkolivegreen were enriched in “translation”, “peptide metabolic process” etc. (Fig. 4a). No significantly enriched GO-BP terms found in brown4, lightyellow and darkmagenta module. KEGG pathway enrichment showed that the DEGs positively associated with the node B were mainly involved in “photosynthesis”, “plant-pathogen interaction”, and “carbon metabolism”; while DEGs positively associated with the trait of the node A were mainly associated with “plant hormone signal transduction” and “Phenylpropanoid biosynthesis” (Fig. 4b). These data showed that these modules and genes positively associated with the trait of node A (blue, salmon4 and brown4) might have crucial roles in controlling Cd accumulation. Among the DEGs positively associated with the trait of the unelongated nodes near the root (node A), we identified that Heavy metal transport/detoxification protein domain containing proteins (OS03G0383900, OS03G0861400, OS10G0506100, OS11G0147500), Copper transporter 6 (OS04G0415600) and Zinc transporters (OS04G0613000 and OS06G0566300) were significantly enriched in “metal ion transport” GO term (GO:0030001), the expression patterns of which mediated these differences in Cd accumulation between the two types of nodes. In addition, their expression levels in node B were lower than those in node A, and were decreased by Cd treatment in the S315 cultivar (Fig. 4c). The gene interaction network analysis results showed that OsDUF246 (OS09G0498800), *OsHlN1* (OS08G0102700) and a conserved hypothetical protein (OS07G0109400) were the core genes in blue module (Fig. 4d), which were highly expressed in node A, but lowly in node B. Accordingly, we speculated that these DEGs were associated with the high Cd accumulation in node A in rice. The up-regulation of them might increase the Cd concentration in node A via sequestering Cd ion by cellular localization and vesicle trafficking.

### DEGs and modules associated with low Cd-accumulative phenotype

**Figure 5:**
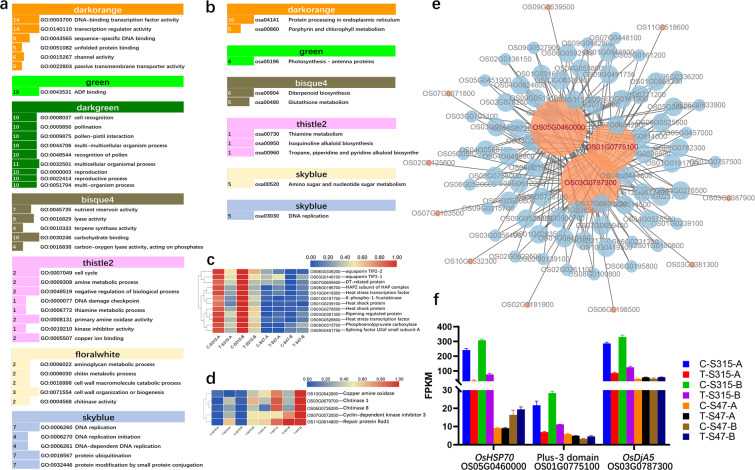
The modules relevant to different rice varieties. (**a,b**) the Gene Ontology biological processes (GO-BP) and KEGG pathways associated with modules associated with high Cd accumulative trait. The number ahead of the items indicates the gene number. (**c,d**) the reads number of several DEGs in the high Cd accumulation positive and negative modules, respectively. e, gene interaction network analysis results of DEGs negatively regulated by Cd stress in S315. f, the expression profiles of key regulators in the interaction network. The bigger the node, the greater the number of connections it has. Nodes with different colors are with different number of connections.

The present results showed that Cd treatment induced more DEGs in the low Cd-accumulative cultivar (S315) versus high Cd-accumulative cultivar (S47). We speculated that these DEGs in S315 cultivar might be responsible for the Cd-induced abiotic stresses. Using WGCNA analysis we found 2 modules (darkorange and green) had positive correlation with S315 (low Cd-accumulative phenotype, cor > 0.5, p < 0.05), while 6 modules (palevioletred3, bisque4, darkgreen, thistle2, floralwhite and skyblue) negatively correlated with S315 (cor < −0.5, p < 0.05). GO enrichment analysis showed that DEGs in the 2 positive modules were significantly enriched in “DNA-binding transcription factor activity”, “transcription regulator activity” and “ADP binding” (Fig. 5a). For negative modules, enrichment analysis showed that DEGs in thistle2 were mainly associated with cell cycle. DEGs in floralwhite were associated with cell wall and chitin metabolic process. In addition, DEGs in skyblue module were mainly associated with BP terms like “DNA replication”, “protein ubiquitination” (Fig. 5a**)**. In the results of KEGG enrichment results, 10 pathways were significantly enriched in these six modules, including protein processing related pathways, photosynthesis, glutathione metabolism, DNA replication pathways (Fig. 5b**)**. The expression profiles of DEGs in the positive modules showed higher reads numbers in S315 cultivar than that in S47 and were decreased by Cd treatment in the S315 cultivar, like heat shock proteins (OS01G0239100 and OS03G0276500), heat stress transcription factors (OS09G0526600 and OS10G0419300), etc. (Fig. 5c). Most DEGs were found in negative modules (darkgreen, bisque4, thistle2, floralwhite, and skyblue), of which DEGs in floralwhite were significantly enriched into GO terms related cell wall, indicating that the cell wall plays a key role in plant resistance to Cd stress. Chitinase 1 (OS06G0726200) and Chitinase 8 (OS10G0542900) were the domain DEGs in cell wall related terms and highly expressed in S47, especially after Cd treatment (Fig. 5d). In thistle2 module, Cyclin-dependent kinase inhibitor 3 (OS11G0614800), Copper amine oxidase (OS07G0572050), and Repair protein Rad1 (OS03G0679700) showed higher expression level in S47 than that of S315 (Fig. 5d). The gene interaction network analysis results showed that *OsHSP70* (OS05G0460000), *OsDJA5* (OS03G0787300) and Plus-3 domain (OS01G0775100) were the key regulators in responding to Cd stress in S315 (negatively regulated by Cd stress in S315, Fig. 5e,f). These data showed that the up-regulation of these genes might be responsible for or at least correlated with the low Cd accumulation. In particular, stress-related signals are active in S47 cells, which is related to the characteristics of high cadmium accumulation in S47.

### DEGs associated with Cd treatment

**Figure 6:**
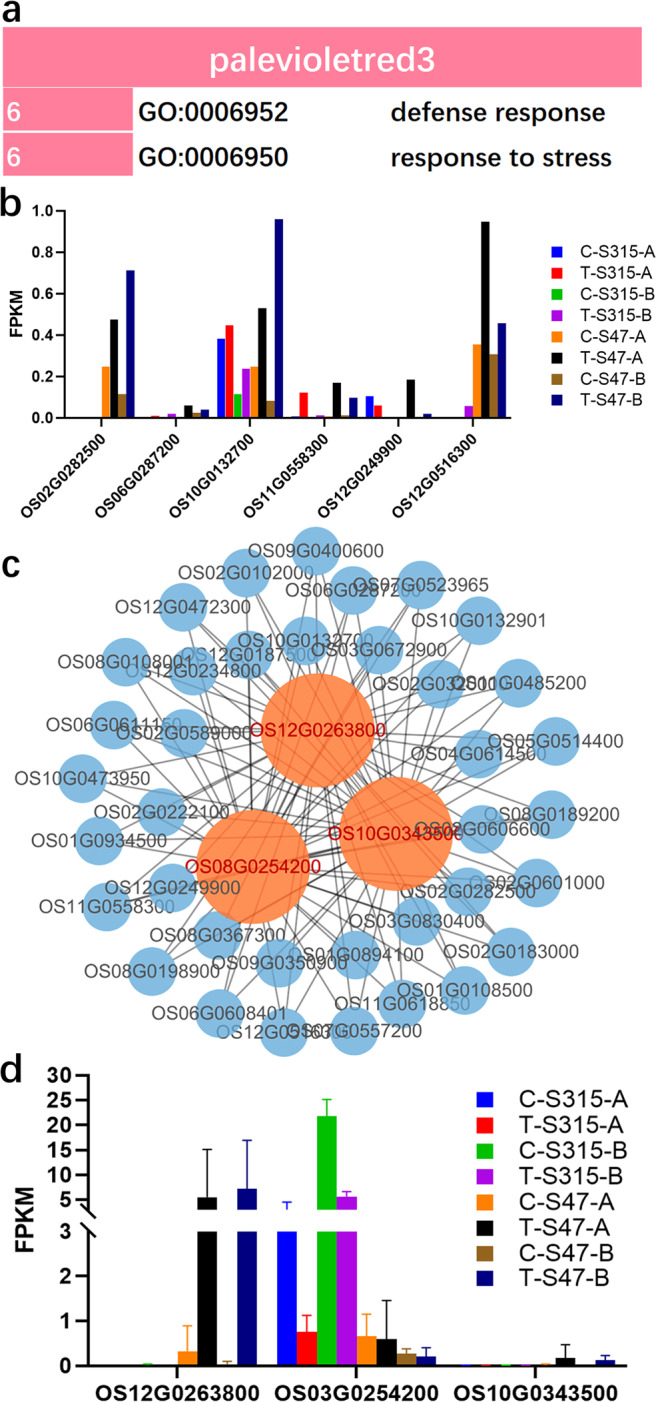
The modules associated with Cd treatment. (**a**) the Gene Ontology biological processes (GO-BP). (**b**) expression of enriched DEGs in GO term “defense response”. The number ahead of the items indicates the gene number. (**c,d**) gene interaction network and the expression profiles of key regulators in the interaction network. The bigger the node, the greater the number of connections it has. Nodes with different colors are with different number of connections.

Our previous analysis showed that only palevioletred3 module associated with the trait of Cd treatment based on the criteria of absolute Cor > 0.5 and p < 0.05 (Fig. 3b). GO enrichment analysis showed that “defense response” and “response to stress” were significantly enriched (Fig. 6a). All the significantly enriched DEGs had low FPKM value (Fig. 6b). The gene interaction network analysis results indicated three key regulators: OS12G0263800 (*OsIRL*, Isoflavone reductase-like protein 1), OS03G0254200 (no annotated information), and OS10G0343500 (Transposase, Fig. 6c), of which OS12G0263800 and OS03G0254200 were highly expressed in S47 and S315, respectively (Fig. 6d).

**Figure 7:**
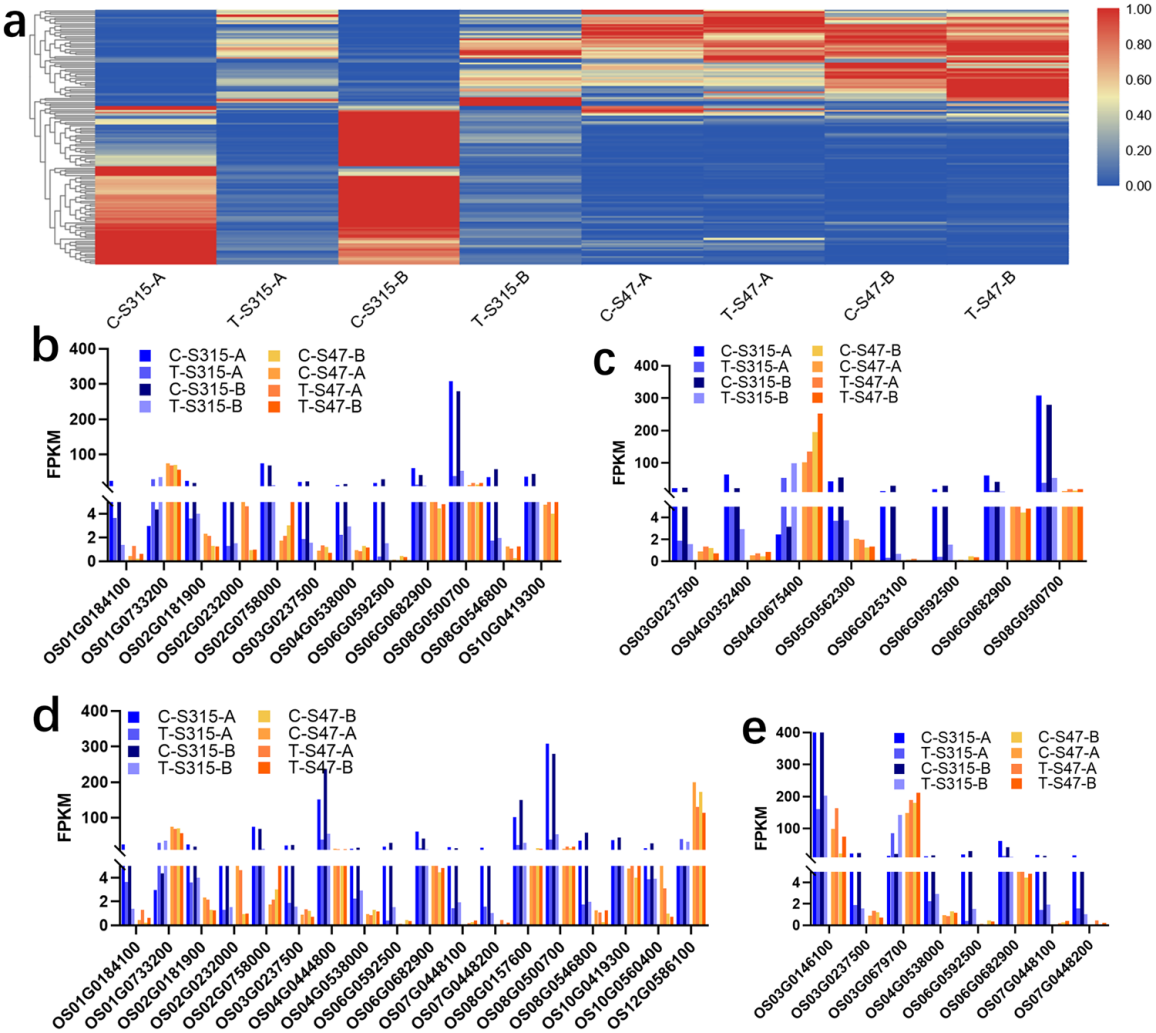
Enrichment analysis results of co-expressed DEGs between S315 and S47. (**a**) heatmap of all the co-expressed DEGs between S315 and S47. (**b** to **e**) expression of DEGs in these significantly enriched GO terms of “response to heat”, “response to temperature stimulus”, “protein folding”, “response to abiotic stimulus”, and “response to drug”.

Because of the great difference between S315 and S47, most of the modules in WGCNA are not significant. Therefore, we analyzed all co-expressed DEGs by GO and KEGG enrichment methods. After screening the co-expressed DEGs induced by Cd treatment, we found the expression patterns were quite different between S315 and S47 (Fig. 7a). The GO enrichment analysis results showed that “response to heat”, “response to temperature stimulus”, “protein folding”, “response to abiotic stimulus”, and “response to drug” were significantly enriched. Most DEGs enriched in these GO terms, including OS08G0500700 (*OsHSP82A*, Heat shock protein 82), OS02G0232000 (*OsHSFC2A*, Heat shock transcription factor 29), OS04G0444800 (*OsFRO1*, Ferric reductase-like transmembrane component family protein), OS07G0448100 (*OsPIP2*, Plasma membrane integral protein), showed higher expression in S315 than S47 in control, which decreased by Cd treatment in S315, but no significant changes in S47 (Fig. 7b–e). No significant enriched KEGG pathways found. These findings showed that genes might have crucial roles in regulating Cd accumulation in rice.

### qRT-PCR validation of selected genes

**Figure 8:**
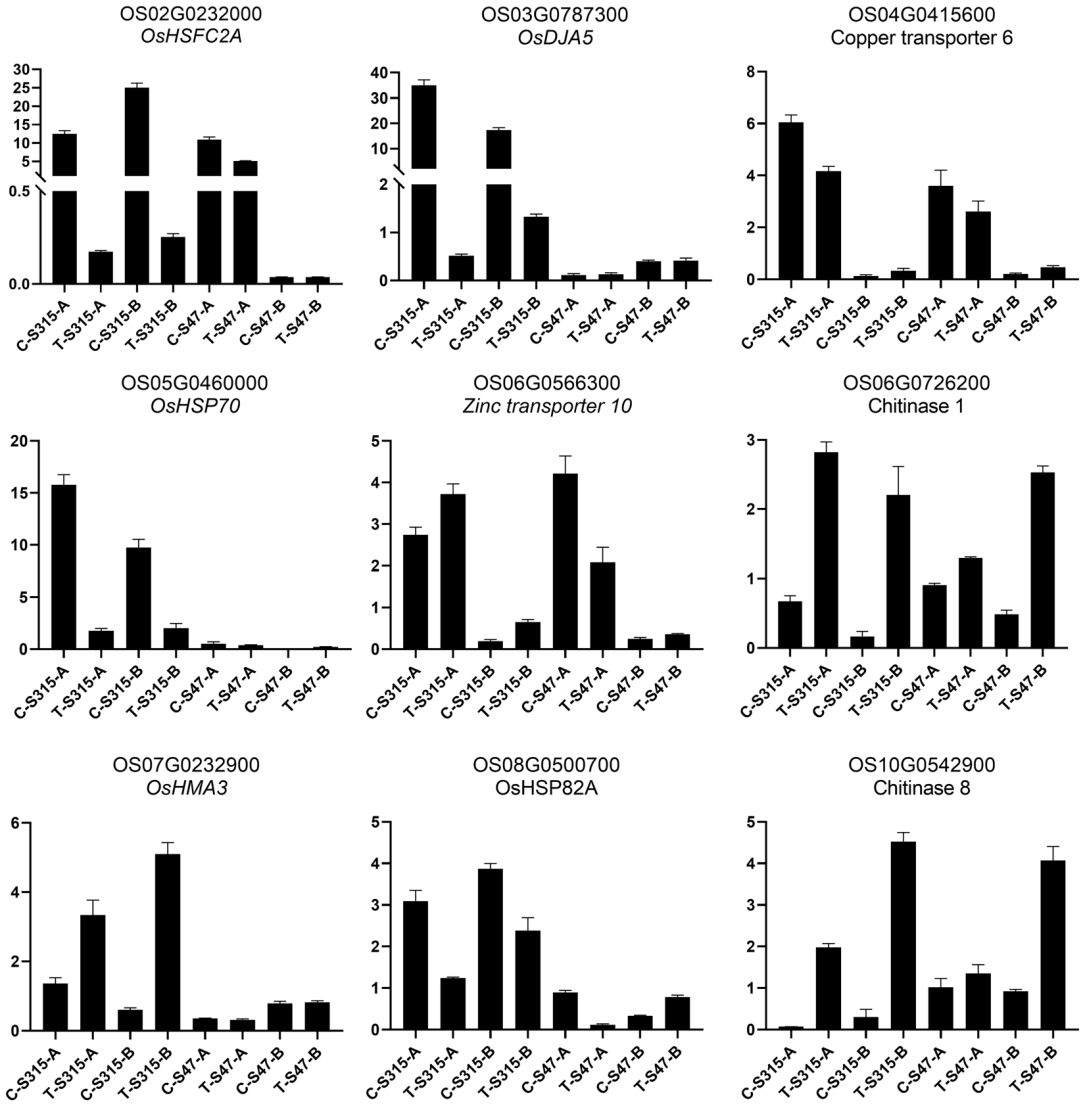
qRT-PCR Validation results. The abscissa shows the genes ID and the ordinate shows the foldchange. Quantification of gene expression was performed by the comparative 2^−ΔΔCT^ method.

The qRT-PCR experiment was performed to confirm the computational analysis results, we selected 9 candidate genes that associated with the Cd accumulation in rice from GO and KEGG enrichment analysis results and WGCNA modules. The results showed that the expression levels of all the genes in qRT-PCR were consistent with RNA-seq. All these 9 candidate genes were significantly changed between different node types, between Cd treatment and control, or between S315 and S47, which demonstrated that these genes were all responsive to Cd (Fig. 8).

## Discussion

The effects of Cd stress on photosynthesis, respiration, and ion transportation in plant have been reviewed in previous studies^17–22^. Our present study demonstrated that the node A of rice had a higher capacity in accumulating and sequestrating Cd ion compared with the panicle node. WGCNA modules and genes associated with the Cd accumulation phenotype and the node A agronomic trait were identified.

Based on the WGCNA, we identified the modules positively associated with Cd treatment, thus the Cd positive modules consisted of upregulated DEGs by Cd treatment. Heavy metal stress has probably triggered the synthesis of many defense proteins as the total protein contents in stressed plants increased^23^. In the present study, chitinase (OS03G0679700 and OS06G0726200) were increased by Cd treatment. Previous studies reported that chitinases are considered to act primarily during pathogen attack by hydrolyzing microbial cell walls^24,25^. Apparently, they might provide advantages for plants during heavy metal stress. Similarly, Meszaros^26^ and Bekesiova^23^ found that heavy metal stress could induce more chitinases in plants. Dana *et al*. reported overexpressing chitinases tobacco showed exhibit enhanced levels of resistance to biotic and abiotic stress^27^. In addition, Wang *et al*. found the up-regulated *AcCHI I* mRNA in response to Cd stress^28^. All these findings further confirmed the important role of chitinases in against Cd stress. Bekesiova *et al*. found that chitinases should be included among defence components that are responsible for inter- as well as intra-species differences in heavy metal tolerance of plants^23^. However, their role and mechanism of action in the plant cells still remain unknown, but are apparently more diverse and complex than expected^23^. WGCNA analysis results also indicated the critical role of *OsHSAP70* and *OsDjA5*. Correspondingly, *OsHSP82A* and *OsHSFC2A* were also found differentially expressed between different nodes and rice varieties. The important roles of *HSF* and *HSPs* (large or small) in plant immunity, growth, defense, and stress responses have been identified^29–32^ and Cd-stress induced *HSPs* expression in rice inhibited Cd-induced damage in plant cell in turns^33^. It has been reported that the overexpression of a small HSP gene enhanced tolerance to abiotic stresses (including heat, drought, abscisic acid, salinity, and cold) in rice^34^. Cai *et al*. showed the silencing of *HSFA1a* in tomato plants blocked Cd stress-induced *HSP* expression, while *HSFA1a* overexpression promoted *HSP* expression^32^. The stable expression of *HSF* and *HSPs* encoding genes, including *OsHSP70*, *OsHSP82A*, and *OsHSFC2A* showed the insensitivity to Cd stress of S47 compared with S315. *HSPs*/molecular chaperones are normally found to be involved in the response to stress^35^. The DnaJ proteins are found in plants and other organisms and are important molecular chaperones, referred to as cellular stress sensors, that are involved in signal transduction, cellular protein homeostasis, and tolerance to multiple stresses in plants^36,37^. Overexpression of the DnaJ-like protein gene could enhance the tolerance to chilling and heat stresses in transgenic tobacco plants^38^. While the decreased *OsDJA5* by Cd treatment in S315 indicated the sensitivity to Cd Stress.

The mRNA expression profiles showed that S315 responded strongly to Cd stress, while S47 responded weakly to Cd stress (Fig. 7a). These differences might mediate lower Cd accumulation in S315. DEGs like OS08G0500700 (*OsHSP82A*), OS02G0232000 (*OsHSFC2A*), OS04G0444800 (*OsFRO1*), and OS07G0448100 (*OsPIP2*) had higher expression level in S315 than that in S47. HSF and HSPs (large or small) play critical roles in plant immunity, growth, defense, and stress responses^29–32^. In rice, Cd-stress induces *HSPs* expression, in turns it inhibits Cd-induced damage in plant cells^33^. Also, PIPs play critical roles in resisting heavy metal stress [49–53]. Accordingly, the expression of these genes was significantly reduced by Cd treatment in S315, but showed small or undetectable changes in S47 cultivar, indicating a relative insensitivity of S47 cultivar to Cd stress. In addition to the DEGs mentioned above, there were many other DEGs involved in the complex Cd stress response process. The difference between the two rice varieties is mainly due to genetic factors, which dominates their different responses to Cd stress.

Stem nodes are responsible for the transport of ions, nutrients and metabolites, and are the central organ from the xylem to the phloem^39^. The different capacities in Cd accumulation between the shoot base and panicle node had been confirmed in previous reports^40–42^. Feng *et al*.^41^ and Fujimaki *et al*.^40^ traced the accumulation and transport of Cd in the rice stem and root and confirmed the gradual decrease of Cd accumulation in rice nodes. Fujimaki *et al*. found Cd was predominantly accumulated in the shoot base (unelongated nodes, then upward transported to the panicle through xylem-to-phloem transfer^40^. Leaf had low Cd accumulation capacity compared with the shoot base and the panicle node^40^. These data showed that the root node might be crucial for the immobilization of Cd. These facts were confirmed by the identification of WGCNA modules associated with the panicle node trait. We identified that the WGCNA modules positively correlated with the node A (named as panicle positive modules) were related to ion transport, photosynthesis and response to stress. However, the modules positively correlated with panicle node (node B) were associated with translation, peptide biosynthetic process, etc. This finding might demonstrate that the node A was crucial for Cd accumulation, cellular localization and upward transport of Cd. Among the DEGs positively related to node A trait, Copper transporter 6, Zinc transporter 10, and some heavy-metal associated proteins were highly expressed in node A compared with node B of the two cultivars. To deal with heavy metal ions, plants, as well as other organisms, have developed a sophisticated homeostatic network to control heavy metal ions uptake, trafficking, utilization, and detoxification or exportation^43,44^. Copper transporter is a main protein that contributes copper ions uptake through the cell membrane^45^. Also, Zinc transporters mediated the uptake of Zinc ions in plant cells^46^. The present study showed the important roles of copper and zinc transporter in different node types. Expression of copper and zinc transporters in node A were significantly higher than that in node B, which is consistent with higher Cd concentration in nodes (Fig. 4c). Therefore, we believe that these genes mediate the accumulation of Cd in node A. Although these genes had been reported to be related to the transport of copper and zinc, they may also play an important role in the transport of Cd. The decreased expression of them in node A after Cd treatment confirmed that these genes were related to the Cd accumulation and transportation in the node A of rice, and might be responsible for the different Cd accumulation mechanisms between the node A and node B. In addition, *OsNramp* genes and *OsHMA3* are both well know Cd uptake transporters^47,48^. Most *OsNramp* transcripts showed no significant changes after Cd stress but were highly expressed in node B compared with node A. Another Cd related transporter *OsHMA3* was significantly increased by Cd treatment in S315 but showed slightl changes in S47. These results indicated that *OsNramp* might mediate the Cd uptake in node B. For S315, the up-regulated *OsHMA3* by Cd stimulation showed a critical role in Cd accumulation.

In addition, some regulators were also identified in the gene interaction network analysis, such as OS12G0263800, OS03G0254200, etc. However, there is a lack information for the association of their expression with stresses. The increase expression of them by Cd treatment might show that their expressions were responsive to Cd stress.

## Conclusions

Our present study demonstrated that Cd stress induced huge changes in gene expression profiles. different rice varieties have different processes and intensities in response to Cd stress. The node A might function as the key tissue for blocking Cd upward transport into the panicle via vigorous processes. DEGs in heavy metal ions transportation (including Copper transporter 6, Zinc transporter 10, and some heavy-metal associated proteins), response to stress (*OSHSPs*, *OsHSFs, OsDJA5*, etc.), and cell wall (chitinase) play critical roles in resisting Cd stress. In addition, many new regulatory factors have been identified in this study, but further experiments would be performed to investigate the mechanism of these DEGs in controlling Cd accumulation in rice and other plants. All the candidate DEGs were verified by qRT-PCR, which will provide new supports for rice breeding.

## Methods

### Plant materials and experimental design

The low Cd-accumulative cultivar “*Shennong 315*” (*O. s. japonica*, short for S315) and high Cd-accumulative cultivar “*Shendao 47*” (*O. s. japonica*, short for S47) were used as plant materials. All the seeds were obtained from the Germplasm Resources Bank of Liaoning Province with the access number of Liaoshendao[2001]No.96 and Liaoshendao[2010]No.235 for S315 and S47, respectively. The experiment was performed in an experimental greenhouse located in Northeast Agricultural University. At three true-leaf stage, rice seedlings were transplanted into greenhouse with conventional density, illumination and fertilization strategy. A total of 60 rice seedlings (including 30 seedlings of S315 and S47 each) were used as plant materials. Each rice variety (30 seedlings) was randomly divided into two pools (15 plants for each), one of which was filled with Cd^2+^-free muddy water (control group, marked as C) and the other one was treated with CdCl_2_·2.5H_2_O (10 mg/Kg) until maturity (Cd treatment group, marked as T). In total, four groups were designed, including C-S315, T-S315, C-S47, and T-S47. The samples of the first node (unelongated nodes, marked as A, Fig. 1), panicle node (marked as B, Fig. 1**)**, and grains were collected at grain-filling stage for Cd content determination with five biological repetitions (three technical repetitions for each). All the stem nodes samples (the first and panicle nodes) were collected for transcriptome analysis and marked as C-S315-A, T-S315-A, C-S315-B, T-S315-B, C-S47-A, T-S47-A, C-S47-B, and T-S47-B, respectively, and three biological replicates were set for each group (each biological replicate contained three individuals). All fresh samples were stored at −80 °C until assayed.

### Determination of total Cd concentrations

To determine the Cd concentration in node A, node B, and rice grains, an atomic absorption spectrophotometer (AAS) was used according to the instructions. Briefly, fresh tissues were shredded, dried and powdered. 100 mg of powder was treated with 1 mL HNO_3_ and was diluted in 20 mL ultrapure water. Standard Cd solution (CdCl_2_) was used as quality control samples.

### Total RNA extraction and mRNA libraries construction

The total RNA was extracted from stem nodes tissues using an RNAprep pure Plant Kit (Tiangen, China). The RNA quality was evaluated using gel electrophoresis and Nanodrop (Thermo, USA). Equal RNA samples from three individuals in each group were pooled to one composite sample (as a biological repetition), and 3 composite samples were prepared in each group (n = 24 samples) accordingly. All the RNA samples were reversely transcribed to cDNA samples using a QuantScript RT Kit (Tiangen, China). The sequence libraries were then constructed using mRNA-seq V3 Library Prep Kit for Illumina (Vazyme, China) according to the manufacturer’s instruction. Library quality was evaluated using Agilent 2100 Bioanalyzer (Agilent Technologies, USA). Finally, an Illumina HiSeq X sequencing platform (a pair-end 2×150 bp mode) was used to obtain sequencing data.

### mRNA sequence data processing

The quality of raw sequencing data (.fastq format) was controlled using the FastQC (version 0.11.5, http://www.bioinformatics.babraham.ac.uk/projects/fastqc/). Low quality reads and adaptor reads were removed from raw data and the clean data were assembled and compared to the reference genome of rice (IRGSP-1.0.28, http://rice.plantbiology.msu.edu/pub/data/Eukaryotic_Projects/o_sativa/) using hisat2 (http://ccb.jhu.edu/software/hisat2). The value of FPKM (expected number of Fragments Per kb per Millions reads) of reads was calculated using Cufflinks (version 2.2.1, http://cole-trapnell-lab.github.io/cufflinks/). Principal component analysis (PCA) and Pearson’s correlation analysis were performed based on the FPKM. The differentially expressed genes (DEGs) were identified using DESeq (http://bioconductor.org/packages/release/bioc/html/DESeq.html)^49^, with the criteria of p < 0.05 and |log_2_(Fold Change, FC) | ≥ 2. Genes with log_2_FC > 2 and log_2_FC < −2 were identified as up- and down-regulated DEGs, respectively. Hierarchical clustering based on the expression profiles of DEGs was presented by pheatmap (version 1.0.10; https://cran.r-project.org/web/packages/pheatmap/index.html).

### WGCNA analyses for DEGs

The R package WGCNA (v1.61; https://cran.r-project.org/web/packages/WGCNA/index.html) was employed for the analysis of DEGs’ co-expression module^50^. The WGCNA parameters of soft threshold power of the adjacency matrix and the criteria of correlation coefficient square of eigengenes were defined according to the approximate scale-free topology preconditions and the criteria of cut-off of ≥30 genes and cut height = 0.15. The adjacency matrix dissimilarity was 0.2. Then, the WGCNA modules (co-expression network) of eigengenes were identified and the networks correlated with agronomic traits were identified with the criterion of stability correlation p ≤ 0.05. The modules with gene significance (Pearson’s correlation coefficient) ≥0.6 for agronomic traits (Cd treatment, Cd accumulation and different tissue nodes) were retained for further analyses.

### GO and KEGG enrichment analysis

The DEGs in modules correlated with the agronomic traits were separately subjected to the enrichment analysis for Gene Ontoloy (GO; http://www.Geneontology.org/) and KEGG (Kyoto Encyclopedia of Genes and Genomes) pathways^51^. Significant GO biological processes (BP) and KEGG pathways were identified with the criterion of p < 0.05.

### qRT-PCR gene expression analysis

qRT-PCR analysis was performed to verify the expression of candidate DEGs. Primers of the candidate DEGs were designed using Primer Premier 5.0 (http://www.PremierBiosoft.com). The 20 μL reaction volume contained 2 μL of diluted cDNA, 0.5 μL of forward and reverse primers (10 μM), 10 μL of 2×POWRUP SYBR MASTER MIX (Thermo, USA) and 7 μL of dd H_2_O. The PCR amplification were performed on an Eppendorf Mastercycler pro PCR System (Eppendorf, Germany) with 95 °C for 5 min, followed by 40 cycles of 95 °C for 15 s, 58 °C for 30 s, then followed by 72 °C for 5 min. The relative quantification was calculated by 2^−ΔΔCT^ method. Three independent biological replicates were designed here.

### Statistical analysis

Statistical analysis was performed using GraphPad Prism 6 (https://www.graphpad.com/support/prism-6-updates/). All experimental data were expressed as mean ± standard deviation (SD), and differences between groups or treatments were analyzed using the unpaired t-test. Differences across tissues were analyzed using one-way ANOVA test. P < 0.05 was set as significant threshold for statistical differences.

### Ethical approval

This article does not contain any studies with human participants or animals performed by any of the authors.

## Supplementary information

Supplementary information.

Supplementary information2.

Supplementary information3.

Supplementary information4.

Supplementary information5.

## Data Availability

The sequencing data were submitted to the SRA database with the Submission numbers SUB6287579.
