# Supplementary material for: Identification of key genes and modules in response to Cadmium stress in different rice varieties and stem nodes by weighted gene co-expression network analysis
Source: Sci Rep. 2020 Jun 12;10:9525. doi: 10.1038/s41598-020-66132-4 (PMC7293223; doi:10.1038/s41598-020-66132-4)
Supplement: Supplementary file 1 — Supplementary information. [file 41598_2020_66132_MOESM1_ESM.docx]

**Identification of key genes and modules related to Cadmium accumulation in Rice stem by weighted gene co-expression network analysis**

Authors: Qi Wang, Xiannan Zeng, Qiulai Song, Yu Sun, Yanjiang Feng, Yongcai Lai


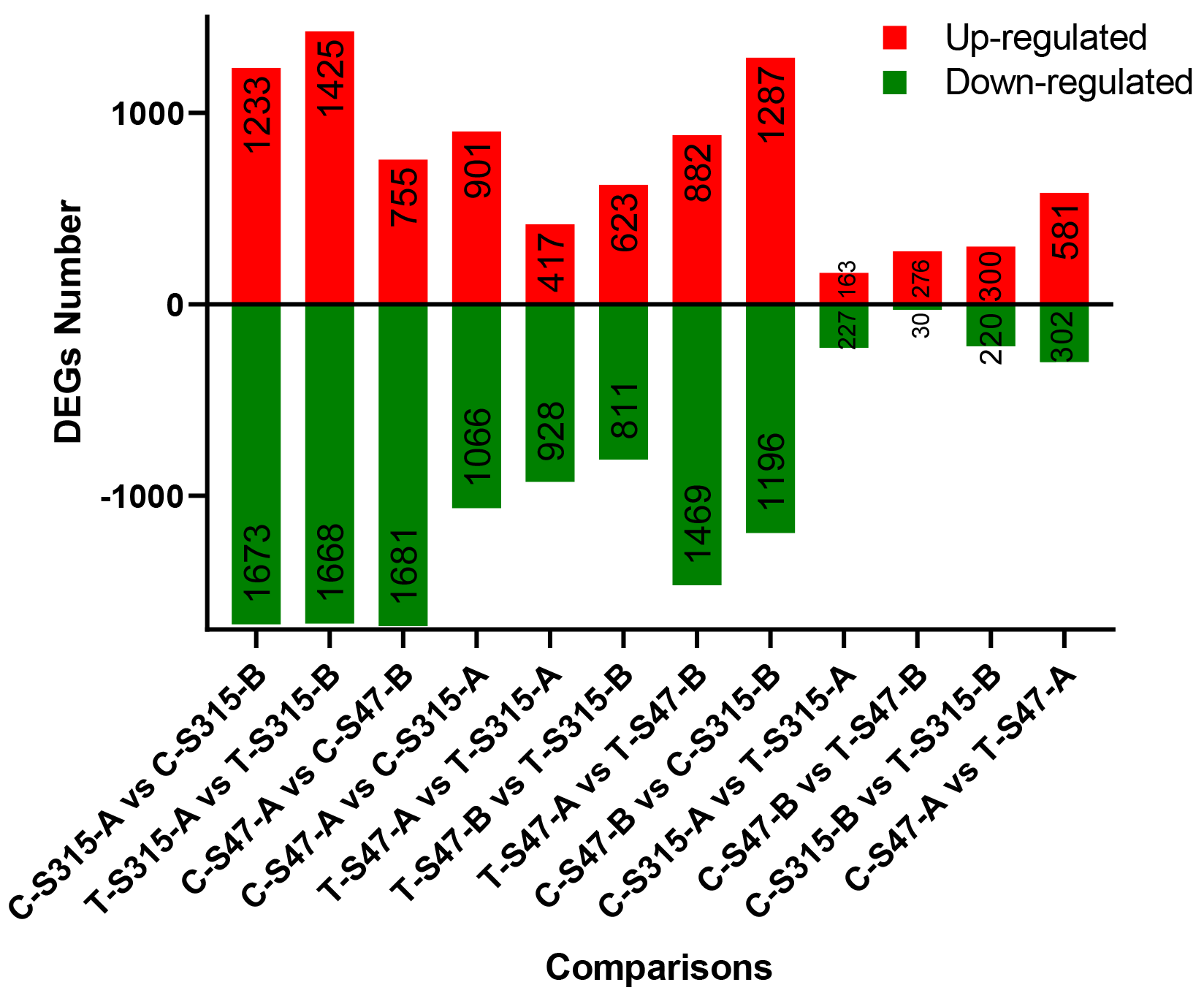


**Supplementary Fig.1 The statistics of the differentially expressed genes (DEGs).** Red and green notes the up- and down-regulated genes.
