# Supplementary material for: Identification of key genes and modules in response to Cadmium stress in different rice varieties and stem nodes by weighted gene co-expression network analysis
Source: Sci Rep. 2020 Jun 12;10:9525. doi: 10.1038/s41598-020-66132-4 (PMC7293223; doi:10.1038/s41598-020-66132-4)
Supplement: Supplementary file 2 — Supplementary information2. [file 41598_2020_66132_MOESM2_ESM.docx]

**Identification of key genes and modules related to Cadmium accumulation in Rice stem by weighted gene co-expression network analysis**

Authors: Qi Wang, Xiannan Zeng, Qiulai Song, Yu Sun, Yanjiang Feng, Yongcai Lai


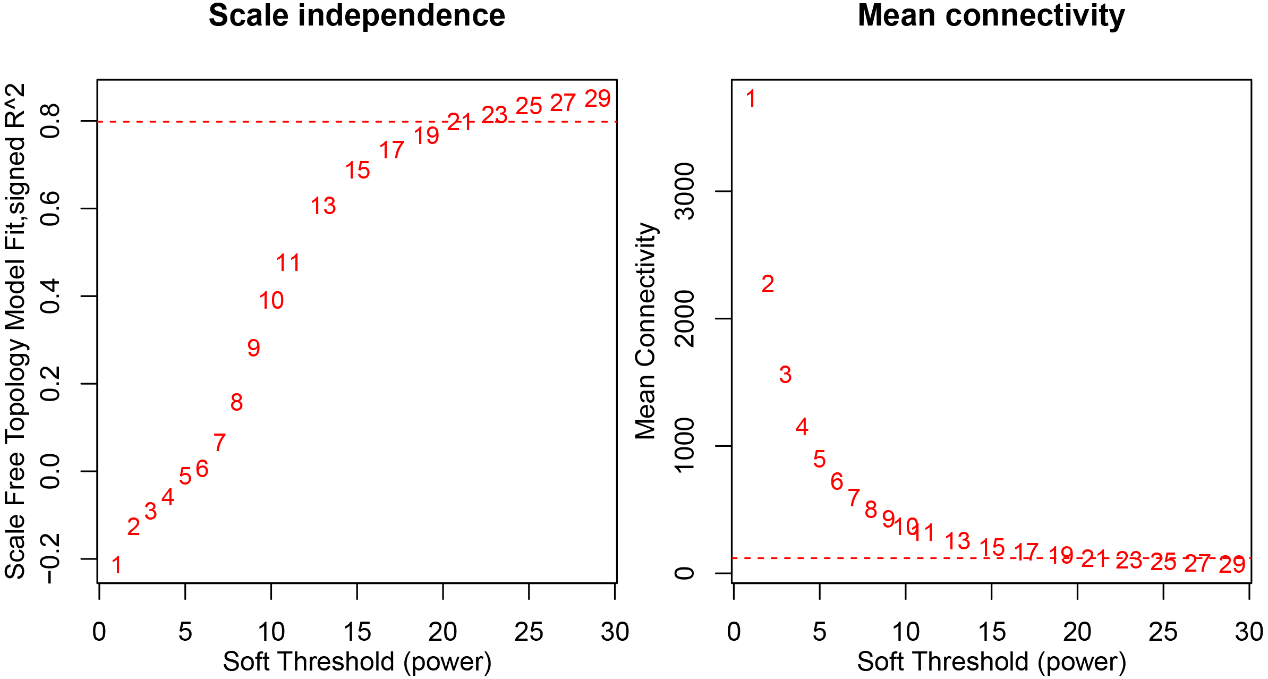


**Supplementary Fig.2. The network topology analysis for adjacency matrix using WGCNA for the DEGs.** The selection of the adjacency matrix soft-thresholding power (left) and mean connectivity of the DEGs (Right) for WGCNA modules.
