# Supplementary material for: Identification of key genes and modules in response to Cadmium stress in different rice varieties and stem nodes by weighted gene co-expression network analysis
Source: Sci Rep. 2020 Jun 12;10:9525. doi: 10.1038/s41598-020-66132-4 (PMC7293223; doi:10.1038/s41598-020-66132-4)
Supplement: Supplementary file 3 — Supplementary information3. [file 41598_2020_66132_MOESM3_ESM.docx]

**Identification of key genes** **and modules related to Cadmium accumulation in Rice stem by weighted gene co-expression network analysis**

Authors: Qi Wang, Xiannan Zeng, Qiulai Song, Yu Sun, Yanjiang Feng, Yongcai Lai

**Supplementary Table 1.** The summary of the mRNA-seq data

| Sample ID | Clean reads (Mb) | Clean Reads Q20(%) | Clean Reads Q30(%) |
| --- | --- | --- | --- |
| C-S315-B_R1 | 53.14 | 96.27 | 92.25 |
| C-S315-B_R2 | 34.40 | 96.04 | 91.81 |
| C-S315-B_R3 | 37.45 | 95.81 | 91.69 |
| T-S315-B_R1 | 55.77 | 96.77 | 92.82 |
| T-S315-B_R2 | 42.62 | 96.48 | 92.44 |
| T-S315-B_R3 | 43.66 | 96.84 | 93.11 |
| C-S315-A_R1 | 47.10 | 96.66 | 92.79 |
| C-S315-A_R2 | 38.01 | 96.53 | 92.61 |
| C-S315-A_R3 | 46.29 | 96.4 | 92.28 |
| T-S315-A_R1 | 50.36 | 97.71 | 92.58 |
| T-S315-A_R2 | 55.30 | 98.75 | 95.42 |
| T-S315-A_R3 | 38.92 | 97.71 | 92.6 |
| C-S47-B_R1 | 38.51 | 97.84 | 92.99 |
| C-S47-B_R2 | 48.25 | 98.81 | 95.66 |
| C-S47-B_R3 | 48.25 | 98.81 | 95.66 |
| T-S47-B_R1 | 54.55 | 97.68 | 92.56 |
| T-S47-B_R2 | 66.64 | 98.72 | 95.38 |
| T-S47-B_R3 | 62.64 | 98.72 | 95.38 |
| C-S47-A_R1 | 55.20 | 97.8 | 92.85 |
| C-S47-A_R2 | 43.40 | 97.78 | 92.85 |
| C-S47-A_R3 | 43.54 | 97.73 | 92.7 |
| T-S47-A_R1 | 42.04 | 97.72 | 92.66 |
| T-S47-A_R2 | 43.25 | 98.74 | 95.46 |
| T-S47-A_R3 | 43.25 | 98.74 | 95.46 |

S47 note Cd high accumulative cultivar “*Shendao 47*”, S315 notes Cd low accumulative cultivar “*Shennong 315*”, “A” indicates the root node, “B” indicates the panicle node
